# Study of the Application of Deep Convolutional Neural Networks (CNNs) in Processing Sensor Data and Biomedical Images

**DOI:** 10.3390/s19163584

**Published:** 2019-08-17

**Authors:** Weijun Hu, Yan Zhang, Lijie Li

**Affiliations:** 1 College of Engineering, Swansea University, Bay Campus, Swansea SA1 8EN, UK; 2 School of Physics, University of Electronic Science and Technology of China, Chengdu 610054, China

**Keywords:** convolutional neural network, images processing, multi-sensor, diabetic retinopathy

## Abstract

The fast progress in research and development of multifunctional, distributed sensor networks has brought challenges in processing data from a large number of sensors. Using deep learning methods such as convolutional neural networks (CNN), it is possible to build smarter systems to forecasting future situations as well as precisely classify large amounts of data from sensors. Multi-sensor data from atmospheric pollutants measurements that involves five criteria, with the underlying analytic model unknown, need to be categorized, so do the Diabetic Retinopathy (DR) fundus images dataset. In this work, we created automatic classifiers based on a deep convolutional neural network (CNN) with two models, a simpler feedforward model with dual modules and an Inception Resnet v2 model, and various structural tweaks for classifying the data from the two tasks. For segregating multi-sensor data, we trained a deep CNN-based classifier on an image dataset extracted from the data by a novel image generating method. We created two deepened and one reductive feedforward network for DR phase classification. The validation accuracies and visualization results show that increasing deep CNN structure depth or kernels number in convolutional layers will not indefinitely improve the classification quality and that a more sophisticated model does not necessarily achieve higher performance when training datasets are quantitatively limited, while increasing training image resolution can induce higher classification accuracies for trained CNNs. The methodology aims at providing support for devising classification networks powering intelligent sensors.

## 1. Introduction

With the rapidly deployment of sensors and actuators, the amount of quantitative data produced by them have been increasing rapidly. To analyze the data and learn the underlying knowledge poses a challenge. Machine learning methods have been widely used in industrial, medication and scientific data processing. Machine learning techniques provide novel ways of analyzing sensor data, so that the meaning of new data can be precisely interpreted based on the learnings from past sensor data. Emerging technologies have demonstrated the integration of machining learning functions with physical/chemical sensors. Machine learning has now become one of mandatory building blocks in a wireless sensor network [1]. There have been many investigations on using the CNN to process data of various sensors. CNN has been used for the retrieval of land surface temperatures from microwave sensors [2]. It has also been utilized in a fall detection system, which consists of many types of sensors such as accelerometers, acoustic sensors, and wearable sensors [3]. Here is a case where CNN technology can be part of a smart sensing mechanism. The traditional imaging sensors (e.g., photodetectors) convert incident light into electronic signals. With the CNN technique, the photo sensing technology could be improved to become close-loop and adaptive, i.e., the electrical signals converted from light can be read by the CNN technique, and then the CNN calculation results can be used to generate a feedback to the sensor to adjust the region of interest, sensitivity, etc. There are more examples that show the seamless link between reported technology and various sensory technologies. Machine learning tasks can be generally divided into supervised learning, unsupervised learning and reinforcement learning. In supervised learning tasks, the algorithm builds a model from a set of data that contain both the input and the desired output. The tasks in this article are supervised learning task with each data specimen having been assigned categories by experts. Classic machine learning methods include support vector machines, boosting, random forest, k nearest neighbor and artificial neural network (ANN). Support vector machines, with appropriate kernel function, can solve non-linear classification problems. With many-fold cross validation, SVM can tackle classification problems involving multiple classes. Using a Gaussian radial basis function as kernel function, the authors of [4] modeled the I-V characteristics of gas sensors using support vector regression (SVR) with temperature and gas concentration as criteria. However, a 3-layer feedforward ANN predicted the I-V characteristics of the gas sensor model with much higher accuracy than SVR when examined with experiment data. Using principal component analysis (PCA) as feature selection method, the authors of [5] employed SVM for classifying multi-sensor data in the prediction of high power laser welding status. Back propagation neural network had been proven to be able to correctly predict the average particle size of TiO_2_ nanosized particle samples from their near-infrared diffuse reflectance spectra [6]. Artificial intelligence paradigms had shown their ability to deal with pattern association, recognition, classification, optimization and prediction tasks in the realm of nanotechnology where many of the systems under study were highly undetermined and several interacting parameters had a strong influence on the results [7].

Deep learning (DL) has the advantage of being sensitive to imperative minute variations while insensitive to large irrelevant variations of the input over classic machine learning methods that rely on linear classifiers on top of hand-engineered features [8]. Deep neural network contains thousands of parameters distributed in the hidden layers, which can be seen distorting input in a non-linear way so that categories become linearly separable by the last layer. Deep learning methods have demonstrated their feature extraction ability for training classic machine learning classifiers such as Adaboost or SVM [9,10,11]. A deep learning (DL) method that utilized feedforward structure rendered the highest prediction accuracy against six other machine learning methods when trained on 271 breast cancer samples, each consisting of measured data of 162 metabolites with known chemical structure [11]. It also successfully learned the top five features that have been proposed as breast cancer biomarkers. The authors of [12] utilized a deep CNN consisting of six one-dimensional convolutional layers with a filter shape of 1 × 3 interspersed with pooling layers for classifying the simulated data which are one-dimensional time-domain signals. The deep CNN achieved higher classification accuracy than that of SVM, while not being susceptible to bias induced by hand-crafted features. In computational mechanics, the authors of [13] built a predictive network based on group method of data handling (GMDH) which is a self-organizing deep learning method for time series forecasting problems without big data requirements. The authors used numerical analysis for tracing a part of equilibrium path, the data of which was then used for training the predictive network. The resulting network demonstrated high accuracy while being much less computationally intensive than conventional approach based purely on numerical analysis. Deep learning has shown its potential in biological image and forensic image classification [9,14,15,16]. The authors of [16] used a feedforward deep convolutional network with interspersed convolutional layers and pooling layers for binary classification of diabetic retinopathy. They also utilized a data augmentation strategy for increasing the limited training image quantity. The authors of [9] carried out the research of applying deep CNN to detecting generative adversarial networks (GANs) generated photo-realistic facial images. They interspersed four dropout layers in the CNN to overcome overfitting resulting from increased network depth. By replacing softmax with the Adaboost classifier in the CGFace model, the newly formed ada-CGFace model achieved classification accuracy that compared very favorably against CGFace model alone when detecting a highly imbalanced dataset containing very a small proportion of computer-generated facial images. AlexNet DNN [10] was utilized as a feature extraction method which was paired with either principle component analysis (PCA)-based or linear discriminant analysis (LDA)-based feature selection for providing training features on which a support-vector-machine based DR classifier was trained. AlexNet DNN-based DR helped classifier achieve accuracy of 97.93% when paired with LDA feature selection, higher than the 95.26% when it was paired with PCA. Using spatial invariant feature transform (SIFT) based feature extraction entailed a classifier of accuracy of 94.4%, confirming the AlexNet DNN based DR feature extraction’s ability.

In this work, we used deep CNN for solving multi-nominal gas sensors data classification task employing a novel data to image conversion mechanism. We then conducted a comparative study on hyperparameter and structure design with a simpler feedforward CNN with dual modules and a state-of-the-art Inception Resnet v2 model. With high resolution color fundus photographs as training dataset, our work can shed light on the influence of changing of hyperparameters or structure on the performance of the resulted CNNs. The characteristics of the mentioned deep learning methods and ours are presented in Table 1.

In the next section, we briefly describe the CNN models that we used. In Section 3, we describe the experiment detail and results of atmosphere pollutant classification with multi-sensor data. In Section 4 and Section 5 we elaborate on the method and results of studying the tweaking hyperparameters and the structure’s influences on CNN performance.

## 2. Computational Methods

### 2.1. Model Design

The prevalent deep CNN models include recurrent neural networks [19], densely connected neural network [20], residual neural network [21], inception v4 [18] and dual-path network [14]. We used a feedforward CNN model with dual modules and Inception Resnet v2 for experiments.

A certain neural network processes a fixed sized input and produce a fixed sized output. The feedforward CNN with dual modules targets input image with a resolution of 66 × 66 pixels or 224 × 224 pixels. It comprises 42 layers, including a simple convolutional layer, three dual-path reduction modules, 10 normal dual-path modules, one pooling layer, one fully connected layer followed by a Softmax function for classification, as shown in Table 2. Batch normalization is attached to every convolutional neuron because it can smooth out the optimization landscape significantly, inducing a more predictive and stable behavior of the gradients, allowing for faster training [22]. Each convolutional unit, after batch-normalized, is connected to nonlinear activation function of rectified linear unit (ReLU) because it generally learns much faster in networks with many layers, allowing training of a deep supervised network without unsupervised pre-training [8].

Inception Resnet v2 [18] is the second variant of the Inception ResNet model. It was introduced with a residual connection to the original inception module, which improves the training speed greatly while retaining the efficiency of realizing optimal sparse structure with dense, readily available components in the inception module [23]. The original inception model is the model powering the well-known GooglNet, which proved its performance in the ILSVRC2014 competition. The Inception ResNet v2 model consists of 22 layers. It achieves a wider instead of deeper network within the same computational budget. A typical dual module comprises one 1 × 1 kernel and one 3 × 3 kernel, whereas a typical module in Inception ResNet v2 model comprises four 1 × 1 kernels, three 3 × 3 kernels. The increase in width of layers for Inception ResNet v2 model enables it to detect a feature regardless of its scale variance [18].

**Figure 1:**
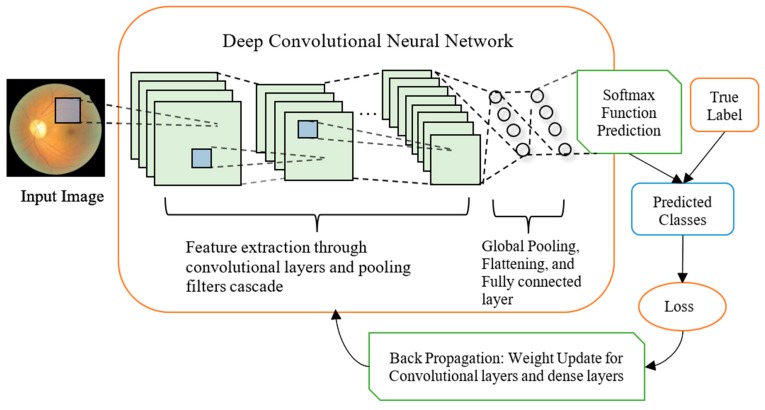
Illustration of classification methodology and deep CNN training process: The input image is analyzed by convolutional filters (Blue squares) which apply to a small receptive field but detecting at each of the image positions and always extending through the whole depth of the image. The resulting output feature maps (Green Squares) are analyzed by convolutional layers or pooling filters at each of their surface positions until being processed by a global average pooling layer which is followed by one fully connected layer. Each green square represents a feature map corresponding to the output for one of the learned features. The convolutional neurons are activated using ReLU function, while the final layer connects to a Softmax function which converts the output into probabilities of the input image belonging to different categories. For training, the true label joins the predicted classes for calculating loss using object function which is cross entropy in our case. The losses are then backpropagated through the network for updating weights of convolutional filters and fully connected layer, where stochastic gradient descent was used for updating weights.

The CNN classification methodology is illustrated in Figure 1. Input images are fed into convolutional layers to be analyzed. A unit in a convolutional layer connects to a small region called receptive field in an input image, but always extending through the whole depth of the image (for a RGB image, the depth is 3). Units in a convolutional layer are arranged in feature maps. The units in the same feature map share the same filter bank. The output of the last normal dual module, designed to be a 336-dimensional feature vector in our work, is flattened and analyzed by a fully connected layer which contains neurons of the number of the classes. The output of the fully connected layer is eventually converted by a Softmax function to probabilities being every classes.

We used t-distributed stochastic neighbor embedding (t-SNE) [24] for reducing the 336-dimension data from the last convolutional layer to three dimensions for visualization. t-SNE has the advantage of being able to preserve local structure of the original data compared to other dimension reduction method such as principle component analysis (PCA). The distance between two samples in t-SNE converted space is decided by the probability of one sample having the other as its neighbor, which equals to the probability of them being neighbor in the original 336-dimension space.

### 2.2. Choosing Hyperparameters

The hyperparameters involved in designing a CNN architecture include the number of kernels, kernel shapes, pooling kernel shapes, learning rate, momentum, and learning rate decay factor. For choosing the number of kernels, it is often the norm to assign the initial layers with fewer kernels and latter layers with more kernels. It is because the neurons in initial layers detect simpler motifs, like small curves and edges, while the latter ones detect motifs that captures more information from larger region of the original image. Kernel shape depends on how much information to capture during convolution. The larger the kernel, the more data will be used in deciding the output from the activation function. Larger kernel size is usually used at initial convolutional layers for quickly sub-sampling input image sizes. Pooling kernel size decides the ability to discard small shifts and distortions and to reduce the dimension of the input. Learning rate determines the footstep size applied during the stochastic gradient descent. Larger learning rates can be beneficial at the early phase of training by allowing to explore different domains of the loss function while decaying the learning over time allows better fine tuning in later stages [14]. Too large a learning rate may cause the object function to miss its lowest point during back propagation while too small a learning rate may cause it to converge to local minimum instead of the global one. Momentum controls how fast the Stochastic Gradient Decent (SGD) process converges. If the objective function during SGD has the form of a long shallow ravine leading to the optimum and steep walls on the sides, momentum needs to be introduced to prevent the SGD from oscillating across the narrow ravine and push the objective towards the optimum more quickly along the shallow ravine [25]. Learning rate decay factor decides the rate at which learning rate decreases for certain epochs.

## 3. Predicting Air Pollutant Type from Apparatus Readings

### 3.1. Data Preparation and Augmentation

The data set used to train our network had been measured using multiple apparatus. Each specimen of air components data includes five parameters, which are doping material, angle, force, air pressure, and ‘in air current’. Together they decide which of the five pollutants—acetone, ethanol, chloroform, toluene, and methanol—the detected air pollutant is. The data measured by the apparatus include 56 specimens. While the remaining four parameters are fixed values, air pressure data was measured as a range. For each measured specimen, we produced the training dataset by keeping four parameters the same while setting the air pressure parameter to values varying in the given range at an increment of 295 Pascal. We then normalized the five parameters independently over all specimens because any value that is larger than 1 will be treated as white color when converting the data into gray-scale images. 

**Figure 2:**
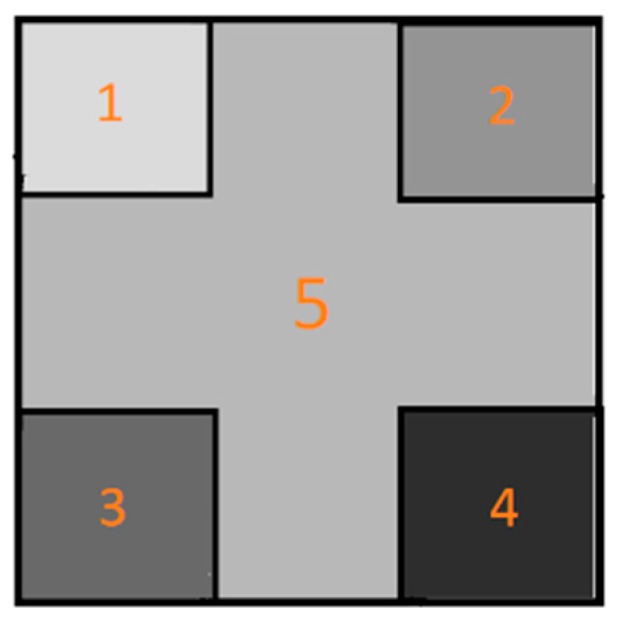
A sample image from training dataset used in training CNN for distinguishing air pollutant. Grey scales of numbered zones are determined by a sample’s five parameters.

For each created specimen, a 64 by 64 matrix was created which comprised five zones that were individually configured with values of the five parameters. The matrixes were then converted to grey scale images. This is because image contains spatial information among the five regions which makes regions of same value in different locations still represent different data which deep CNN can distinguish. Conventional machine learning methods do not consider spatial information, since the image will be flattened or have features extracted before classification. The schematic image that embeds all five of the parameters is illustrated in Figure 2. A similar method of converting data into HSV data and further into RGB images was used to transforming the quantum Monte Carlo sampling data for building a fermionic phases distinguishing method based on convolutional neural network [26]. The squares marked 1, 2, 3, and 4 represent the first four parameters while the square marked 5 represents the most decisive parameter, the ‘in air current’ data. The whole square is 64 by 64 pixels in size with small squares marked with 1, 2, 3 and 4 each being 20 by 20 pixels in size. With this training dataset creating mechanism we obtained 537 images. We then created another training dataset of 993 images by reducing the air pressure parameter value increment to 147.5 Pascal. We trained two CNN instances separately on the two datasets.

### 3.2. Training Process and Results

The ratio of training set over validation set is set at 0.9. We used the feed forward CNN model with dual modules. We initialized the weights in the CNN by imparting them random values from normal distribution using Xavier method, which keeps the scales of gradients roughly the same in all layers. Such weight initialization was also used in the training of feedforward CNN for DR classification.

With batch size of 56 and training for 200 epochs, the best validation precision appeared during training on the 537 images set is 0.892857. Training on the 993 images data set for 200 epochs entails highest validation accuracy of 0.9375. It is 0.04464 higher than the previously one. The increment indicates that the validation precision of trained network is positively related to the size of the training dataset.

**Figure 3:**
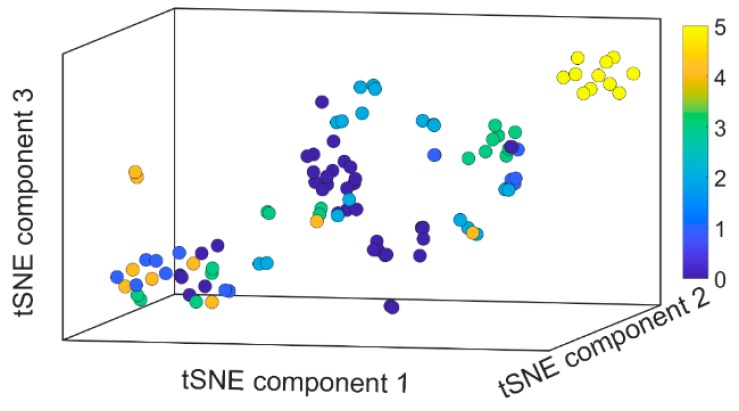
3-dimensional t-SNE visualization of the result of air component experiment validation set in activation space representation. Class 0 to 4 represent acetone, ethanol, chloroform, toluene, methanol, respectively. Finally, class 5 represents a new undefined ingredient apart from the defined five types of ingredients.

The last normal dual module in the trained CNN produces a 336-dimensional feature map for each validation image. All the feature maps of validation set images form an activation space. The activation space is transformed into a 3-dimensional representation space by t-SNE algorithm in MATLAB. The visualization of it is shown in Figure 3. The circles are colored according to their respective classes. Because we measured current passing through different doping material in different air pollutant, there is minimal internal correlation among different measured data specimens. This is reflected in the t-SNE visualization that circles from different classes are not arranged according to the sequence of their class numbers. Even circles belonging to the same class can appear in different locations in representation space. The large distance between circles group in t-SNE visualization indicates that their represented specimens share very few features with each other. The trained network also learnt a new class 5 that was not known during training data preparation, in an unsupervised manner. It could be data specimens that represent the air itself. The trained deep CNN can be used for classification of apparatus data considering the classification accuracy of 0.9375.

## 4. Classification of DR Fundus Images Using CNNs

We then studied the classification of color fundus images in diabetic retinopathy (DR), which is the leading cause of blindness in the working-age population of the developed world. The aim is to investigate the application of CNN in DR phases classification. We also modified the CNN model for training on higher resolution images by firstly making the network reduce the image dimension quicker in the early layers. We then added two more reduction dual-path and four more normal dual-path modules to the feedforward network and compared the results.

### 4.1. Data Set Preparation

The training samples and validation samples that we use were released by EyePACS, LLC [27]. The DR dataset contains 35,126 high-resolution human fundus images for training and 53,576 equally high-resolution raw fundus images for testing. The fundus raw images can be categorized into five different phases of disease progression according to their severity. The dataset comprises images acquired from both left and right eyes of the patients. Table 3 displays the fundus images distribution in the DR dataset. The DR fundus raw image data set is accompanied by an excel file which stores the DR phases numbers to their corresponding images which were assigned by qualified clinicians. The raw images are of sizes that are in the thousands by thousands of pixels range which were too large to train on. Therefore, we needed to resize them to smaller uniformed sizes in preparation for training.

### 4.2. Training a CNN Instance with Feedforward Model of Dual Modules

**Figure 4:**
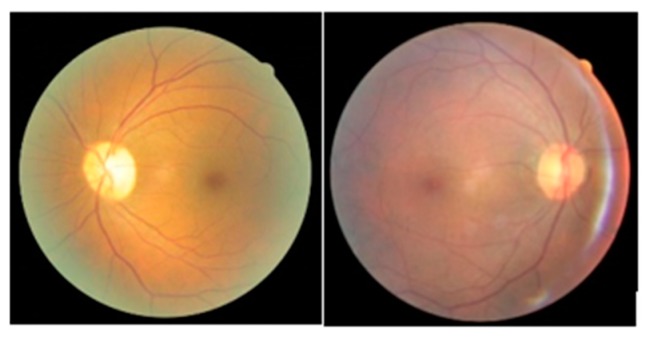
Two randomly picked fundus images from the training data set that feature oppositely oriented retinas.

Firstly, we resized the fundus raw images to have their shorter borders to be 64 pixels and then crop the central squared regions out of them. This ensured that the eyeball’s aspect ratio remained unaltered. Apart from resizing we did not perform any image post processing or enhancement because CNN’s biggest benefit is being able to learn information directly from raw images. We notice that the fundus images can be of opposite orientations as illustrated in Figure 4. Even among solely left eye images, there are significant proportions of retinas that are of opposite orientations to others. But we decided not to flip the images to make them align along the same direction. The consideration was two-fold. Firstly, the generic patterns of diabetic retinopathy symptoms, like blood vessel visibility and haze region, should be shared by both oppositely facing retina images. Secondly, the neural network will be able to associate fundus images of both sides to professionally classified labels which have practical usage in real life. The sample images are arbitrarily assigned to either training set or validation set by the sample preparation program at a ratio chosen as 0.9 between training set and validation set.

**Figure 5:**
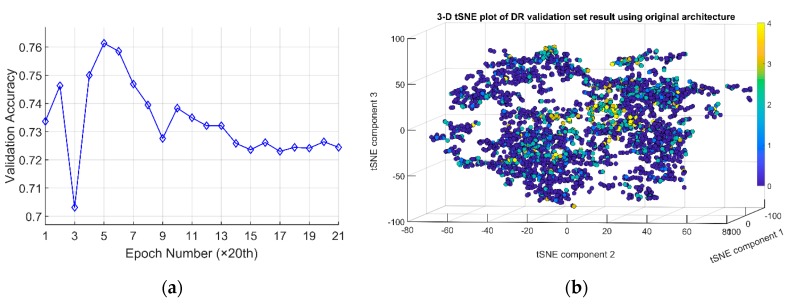
(**a**) validation accuracies recorded for every 20 epochs over 400 epochs. (**b**) t-SNE visualization of validation set in activation space representation by the trained CNN with feedforward structure with dual modules. The arrangement of circles of class 0 to 4 in 3-dimensional space reflects the correct sequence of disease progression.

The hyperparameters in the training of CNN in DR phases distinguishing were chosen as follows. Learning rate was 0.01. Learning rate decaying factor was chosen as 0.1. Momentum was chosen as 0.9. Weight decaying rate was chosen as 0.0005. The training program was designed to run for 400 epochs. An epoch is one round of optimization. The highest validation accuracy recorded during the training process of the trained CNN is 0.763068 at epoch 95. After epoch 95, the validation accuracies of remaining epochs start to gradually decrease. It falls back to around 0.72 at the end of the training (Figure 5a). This shows that training for prolonged epochs does not translate into increased validation accuracy of the network. So long as the number of epochs is enough to allow it to reach the maximum value, there is no need to train an extensive number of epochs. The t-SNE visualization of the activation space representation by the original CNNs is shown in Figure 5b. Even though segregation of dots representing instances from classes 0 to 4 is present, the continuity information among classes 0–4 is not evident.

### 4.3. Training a CNN Instance on Higher Resolution Samples

Each CNN structure is specifically designed for a certain image resolution. Otherwise the network is unable to properly reduce the dimension of the input image for global pooling layer to process, leading to too many variables in the flattened feature vector. When we increased the resolution of the training data record, we modified our CNN architecture accordingly. We uniformly resized the data record size to 224 by 224 pixels, a resolution that should retain more useful information and learnable features than that of retinas images of a resolution of 66 by 66 pixels.

Firstly, we kept the structure of the original deep CNN structure unchanged yet making the first simple convolutional layer more aggressive at reducing the dimension of the input images. The kernel architecture is shown in Table 2 which contains sizes and numbers of kernels in each constituting module. We modified the first simple convolutional layer by increasing the convolutional kernel size from 3 × 3 pixels to 9 × 9 pixels and the stride from 1 × 1 pixel to 3 × 3 pixels. Similar configuration strategy has been employed in the inception net structure design [28]. This increase in convolutional kernel size will allow the kernel to analyze a larger region of pixels on the input raw image in 1 round of convolution. This makes each pixel in the output feature map of the convolutional layer incorporate more information from the raw image. Meanwhile, the enlargement of stride for the kernel effectively reduced sizes of the outputs. 

**Figure 6:**
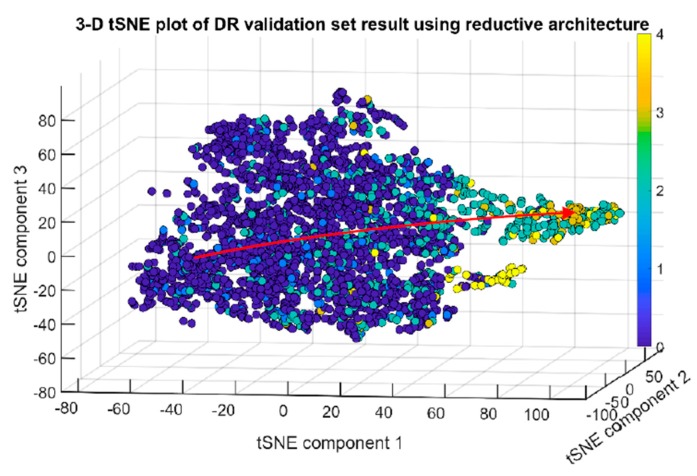
Reconstruction of disease progression in diabetic retinopathy by CNN with reductive first convolutional layer that was trained on high resolution images. The red arrow indicates the inferred disease progression sequence among dots representing class 0 to 4.

The training was set to run for 100 epochs. The highest validation accuracy of 0.809106 appeared at the 90th epoch. It decreased slightly afterwards but remained higher than 0.8 over the next 10 epochs. Training the network with reductive first convolutional layer had proven to be very effective at consolidating the information of the raw images. The training program can process a batch size of 68 images at a speed of 130 images per second. Training for 100 epochs took only approximately 6 h. According to the label definition, label 0 to 4 represent no diabetic retinopathy (DR), mild, moderate, severe, and proliferative DR respectively. The 3-dimensional t-SNE visualization of the validation set in activation space representation is shown in Figure 6. 

Samples with label 0 to label 4 are aligned correctly according to the disease progression sequence from healthy (label 0) to severe (label 3). The dots representing proliferative (label 4) do not follow the progression curve that are formed by dots labelled 0 to 3. Meaning that proliferative DR images have more features in common with those of medium (3) than severe (4) DR. Class 5 is an inferred class. The dots representing class 5 scatter among the main body of dots that represent class 0, 1 and 2. The red arrow represents the inferred direction of disease progression represented by t-SNE visualization of activation space of the validation image set by the trained CNN.

When the input image is a square, the feature map dimension produced by the kernel can be calculated by Equation (1):

(1) $$W_{out}=\frac{W_{in}-K+2P}{S}+1$$

where $W_{in}$ and $W_{out}$ are widths of input image and output image respectively. K is the kernel width. P is the padding width. S is the stride width. The replication of filters along the width and height of the input volume allows features to be detected regardless of the location in a visual field, thus realizing the property of translational invariance.

## 5. Analyzing the effects of deepening CNN architecture

### 5.1. Experiment Details

To study the influence of the depth of a neural network architecture, four more normal dual-path modules and two more dual-path reduction modules were inserted into the neural network. They were inserted behind the last normal dual-path module and before the global pooling layer in the original network structure (Table 4). The added normal dual module contains 224 (1 × 1) kernels and 192 (3 × 3) kernels, while the former highest (1 × 1) kernel number and (3 × 3) kernel number are 176 and 160 respectively. This should enable the trained CNN to learn a wider range of global features from the training data set. A reduction dual module reduced the size of the output feature map to a quarter of the original size for every other two normal dual-path modules. The reduction was realized by making the pooling kernel and (3 × 3) kernel slide at a stride of 2 × 2 pixels for one time of calculation along the width and the height direction of the input data. The reduction dual modules ensure that the output from the normal modules can be small enough that global pooling layer can finally down sample it to desired dimension to be flattened.

For preparation of training and validation data set, we have uniformly resized them to a resolution of 224 by 224 pixels during training record file preparation. The ratio between training dataset over test dataset was chosen as 0.9. The batch size was set as 7.

### 5.2. Training Results Analysis

**Figure 7:**
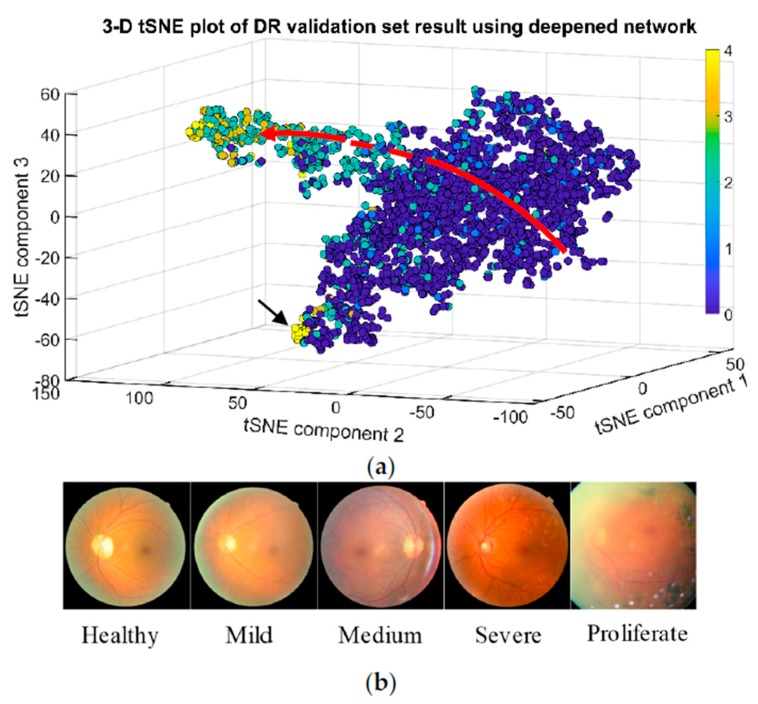
Reconstruction of disease progression in diabetic retinopathy by the deepened CNN. (**a**) tSNE visualization of validation image data set in activation space representation, colored according to corresponding disease phase. (**b**) Randomly picked repetitive fundus images that from each phase.

Training for 100 epochs took 23 h. The highest validation accuracy of 0.813068 among the 100 epochs had been recorded, which appeared at epoch number 93. As the t-SNE visualization (Figure 7a) shows, locations of circles from classes 0 to 3 in 3-dimensional representation of activation space exhibit the correct order of DR disease progression. The inferred classes arranging sequence displayed by the visualization is indicated by a red arc (Figure 7a) with the arrow pointing at the progression direction. The dashed line in the middle of the arc represents an area where circles are scarce. The scarcity of circles should not be interpreted as a lack of samples acquired that belong to this missing phase of disease progression. Rather, it should be interpreted as the phenomenon that fundus images of phases 1 and 2 share very few common features in the 416-variable feature maps produced by the CNNs. This translates to the fact that there are big changes in retinas when the disease progresses from mild phase to medium phase, which is reflected in Figure 7b.

Notice that circles representing phase 4 do not appear behind end of circles of phase 3. Instead they aggregate as a cluster (pointed by a black arrow in Figure 7a) appending to the end of clusters consisted of circles representing phase 2. This indicates that fundus images of prolific phase share more features with images of normal and mild phases instead of with those of severe phase. Nevertheless, circles of class 2 and class 3 are still well separated from the circles of class 0 and class 1, making detecting DR in its early stages using CNN desirable.

### 5.3. Revised Deepened Architecture

Upon inspecting the 416-variable data in the feature file produced by the trained CNN described in the previous section, it was found that there were multiple columns of data consisting of completely zeros. The activation function that we use for constructing a neuron is rectified linear unit (ReLU), which is written as:

(2) f(x)=max(0,x)

where it outputs 0 for input of negative value. This means that the neurons in the feature maps responsible for the variables with 0 values have not made ReLU ‘activate’ at all, leading to the global pooling layer producing 0 values. We count the number of variables that are all zeros as 107, leaving the number of non-zero variables as 309. This number of variables that are not all zeros was less than that were in the feature file produced by the original CNN and the CNN with reductive first convolutional layer. We noticed that the last two normal dual-path modules of the deepened CNN (Table 4.) leveraged on input data size of only 7 × 7 (width × height). For comparison, the last two normal dual-path modules in the original CNN and the CNN with reductive first convolutional layer receive feature maps that are 8 × 8 and 10 × 10, respectively. We assume that the shrink in input data size for the final normal dual-path modules was the reason for some kernels failing to learn any feature, thus outputting all zeros for all validation set images. We then reduced the kernel numbers in the added normal and reduction dual-path modules (Table 5) to find out the optimal kernel number to make sure that all kernels can learn features from the 7 × 7 resolution input. The training ran for 100 epochs, taking 15 h. The highest validation accuracy (0.819294) recorded appeared at epoch number 100. The meaningful variables number is 295 from the 336 total variables in the feature map. Despite the total useful kernels number being smaller than that of the previous deepened CNN, the empty kernels number had dropped by half (38 against 107). We conjectured that the size of the input image does limit the number of features a convolutional layer can learn from. The increase in kernel numbers of latter global convolutional layers cannot continually improve validation accuracy when they have captured all learnable global features.

### 5.4. Training Using Inception Resnet v2 Model

We built and trained a network based on the Inception Resnet v2 model using TensorFlow 1.13 on the same hardware. The training details are as follows. The whole dataset of 35,125 images was divided into training and validation set by a ratio of 0.9. They were resized to 299 by 299 pixels using bilinear filtering method and packaged into 10 TFRecord files, five each for the training and testing datasets. During training, we further resized the images to 240 by 240 pixels and fed them to the network with a batch size of 32. In terms of hyperparameters, we used an initial learning rate of 0.01. Learning rate decayed for every two epochs at a learning rate decaying factor of 0.94 using exponential decaying method. Loss function was chosen as Softmax cross entropy. The optimization method was stochastic gradient descent. We use a momentum of 0.9 for propelling gradient descent. Training Inception ResNet v2 model for 100 epochs costed approximately 42 h which is eight times of that of the reduction dual-module network. It reflects the fact that the Inception ResNet v2 contains more weights. It is certainly slower to train than all variants of the feed forward model with dual modules.

**Figure 8:**
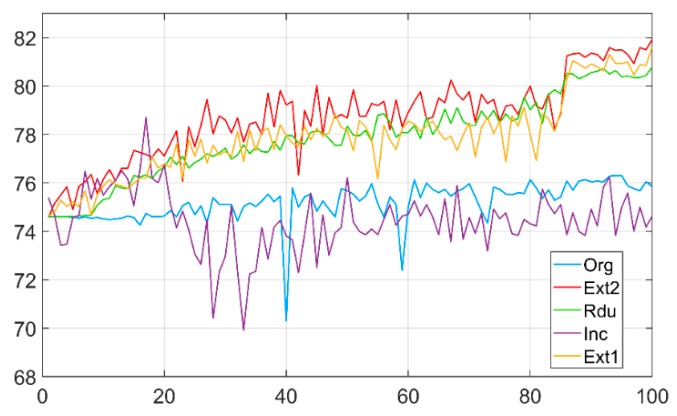
Validation accuracies of five different CNNs. ‘Org’ represents original feedforward CNN; ‘Ext2’ represents revised deepened CNN; ‘Ext1’ represents deepened CNN; ‘Rdu’ represents reductive CNN; ‘Inc’ represents Inception Resnet v2.

The highest validation accuracy among the 100 epochs was 78.708%, appearing at epoch 17. The validation accuracies then fell to its lowest point before rising to a steady 74% as shown in Figure 8. The accuracy curve of the reduction dual module network stays above that of the Inception Resnet v2 model after around epoch number 20 at an increasingly wider gap. We contribute this reduction in accuracy to the fact that the fundus images have been uniformly scaled with similarly sized retinas in the center, which make the differently sized filter banks in an inception module redundant since they are designed to detect features at different scales simultaneously [23]. We also recorded the training accuracy for every 100th batch. We found that since epoch number 40, the training accuracies had remained constantly above 99%, which were much higher than the validation accuracies of the same epochs. We deduced that the trained model had encountered overfitting problem, likely due to the limited size of the training dataset. The Inception ResNet v2 model was originally trained on ImageNet database which includes millions of images of resolutions of 240 × 240 pixels, while our dataset constitutes 35,000 images.

### 5.5. Comparison of CNNs of Five Different Architectures

We trained our networks on a single Nvidia GTX1070Ti-powered PC. The best validation accuracies from various CNN architectures in DR phase classification are listed in Table 6. Both the deepened architecture and reductive architecture have achieved validation accuracies that are over 4% higher than the original feedforward architecture during training the same number of epochs. This proves that training with higher resolution data can improve the accuracy of the CNN. Higher resolution training data provides more abundant information for the CNN to leverage on, resulting in learned filters that are more accurate at classification. The deepened architecture has achieved an accuracy that is 0.5245% higher than the reductive architecture. Within margin of error, they are equivalent. It can be concluded that adding more convolutional layers alone will not improve the accuracy any further after it reaches certain depth [21]. The revised deepened architecture, with fewer kernels in added dual modules, also achieved similar accuracy, within margin of error, to the deepened architecture (Figure 8. This could be attributed to the meaningful global features that can be learned from the output of the earlier layers are limited. Therefore, increasing kernel numbers cannot continually improve accuracies of CNNs.

Despite being able to take advantage of the efficiency of mimicked sparse structure with dense, readily available components, Inception Resnet v2 lost the best performing to the simpler feedforward networks in classification quality by a margin of 7%. It also took the longest time to train. Just to benchmark our model with the online leader board on the DR recognition, our best result of classification accuracy of 81.9% sits in the top 10 places of the current leader board.

## 6. Conclusions

In this article, we employed deep CNN for analyzing multi-sensors data of atmospheric pollutant measurement and classification of DR progression phases for color fundus images. A data transformation method that can embed multi-variable data into an image for training deep CNN classifier was proposed. It capitalizes on the fact that the convolutional unit scans the image from top to bottom and left to right, allowing different regions of the same grey scale value on the same image hosting different information. Because deep CNN can classify image at a rate of hundreds of images per second, it can undertake real time multi-sensor data classification task. Through experiments with CNNs distinguishing DR phases for fundus images, we learned 5-fold lessons as follows. Firstly, the global features which can be learned from a given image by deeper convolutional layers are limited. Secondly, configuring more layers in a CNN architecture or more kernels in latter convolutional layers will not improve CNN validation accuracy continually. Thirdly, training on higher resolution images induce higher validation accuracy for the trained network. Fourthly, for training CNNs on high resolution data or large amount of data, designing architectures with a first convolutional layer that have larger kernels and bigger stride can help sub-sample the input image more effectively while retaining the information embedded in the image. Fifthly, training with Inception ResNet v2 model shows that when tackling object recognition problems in specialized realm, where objects of interest are uniformly scaled in images, the variously sized filter banks can be rendered redundant since decisive features are of the same scale. Three CNNs trained on high resolution image achieved 5-class validation accuracies exceeding 80%, which can help in real world diagnosis. For solving the overfitting problem arisen with deep CNN, adding dropout layers to the CNN structure should help.

## Figures and Tables

**Table 1:** Comparison of Deep Learning Methods.

Methods	Characteristics
Deep feedforward convolutional neural network [17]	Learning a hierarchy of features including simple curves and edges to global motifs from raw images. Sensitive to crucial minute details yet insensitive to large irrelevant variations in image.
Group method of data handling [13]	Self-organizing deep learning method for time series forecasting problems without requirement of big data
Ada-CGFace framework [9]	Uses Adaboost classifier in place of softmax. Contains dropout layers for avoiding overfitting and trained using adaptive moment estimation instead of stochastic gradient descent.
Deep CNN with dual modules (our method)	Has certain level of scale invariability to target object. 1 × 1 convolutional kernel induces small computational cost.
Inception Resnet v2 [18]	Residual connection improves training speed greatly. Inception is computationally efficient. It can abstract features at different scales simultaneously

**Table 2:** The CNN architecture modified for training on higher resolution images.

Module Name	Kernel Size (Width × Height × Channel), Number and Stride	Output Size
Input Raw image	N/A	(224 × 224 × 3)
Simple Convolution	9 × 9 × 3 Conv (96 stride 3)	(74 × 74 × 96)
Normal dual-path modules 1	1 × 1 × 96 Conv (32 stride 1), 3 × 3 × 96 Conv (32 stride 1)	(74 × 74 × 64)
Normal dual-path modules 2	1 × 1 × 64 Conv (32 stride 1), 3 × 3 × 64 Conv (48 stride 1)	(74 × 74 × 80)
Dual-path reduction module 1	3 × 3 × 80 Conv (80 stride 2), 3 × 3 Max pooling (1 stride 2)	(37 × 37 × 160)
Normal dual-path modules 3	1 × 1 × 160 Conv (112 stride 1), 3 × 3 × 160 Conv (48 stride 1)	(37 × 37 × 160)
Normal dual-path modules 4	1 × 1 × 160 Conv (96 stride 1), 3 × 3 × 160 Conv (64 stride 1)	(37 × 37 × 160)
Normal dual-path modules 5	1 × 1 × 160 Conv (80 stride 1), 3 × 3 × 160 Conv (80 stride 1)	(37 × 37 × 160)
Normal dual-path modules 6	1 × 1 × 160 Conv (48 stride 1), 3 × 3 × 160 Conv (96 stride 1)	(37 × 37 × 144)
Dual-path reduction module 2	3 × 3 × 144 Conv (96 stride 2), 3 × 3 Max pooling (1 stride 2)	(19 × 19 × 240)
Normal dual-path modules 7	1 × 1 × 240 Conv (176 stride 1), 3 × 3 × 240 Conv (160 stride 1)	(19 × 19 × 336)
Normal dual-path modules 8	1 × 1 × 336 Conv (176 stride 1), 3 × 3 × 336 Conv (160 stride 1)	(19 × 19 × 336)
Dual-path reduction module 3	3 × 3 × 336 Conv (96 stride 2), 3 × 3 Max pooling (1 stride 2)	(10 × 10 × 432)
Normal dual-path modules 9	1 × 1 × 432 Conv (176 stride 1), 3 × 3 × 432 Conv (160 stride 1)	(10 × 10 × 336)
Normal dual-path modules 10	1 × 1 × 336 Conv (176 stride 1), 3 × 3 × 336 Conv (160 stride 1)	(10 × 10 × 336)
Pooling layer	10 × 10 Average pooling (1 stride 1)	(1 × 1 × 336)
Flatten	N/A	(336 × 1)
Fully connected layer	Hidden nodes (5)	(5 × 1)
Softmax layer	N/A	(5 × 1)

**Table 3:** DR fundus images distribution.

Class	DR Classification	No. of Images	Percentage (%)	Imbalanced Ratio
0	Normal	25,810	73.48	1.01
1	Mild NPDR	2443	6.96	1.84
2	Moderate NPDR	5292	15.07	1.26
3	Severe NPDR	873	2.48	2.76
4	Proliferative DR	708	2.01	2.89

**Table 4:** The deepened CNN architecture with 6 more modules inserted.

Module Name	Kernel size (Width × Height × Channel), Number and Stride	Output Size
Input Raw Image	N/A	(224 × 224 × 3)
Simple Convolution	3 × 3 × 3 Conv (96 stride 1)	(224 × 224 × 96)
Normal dual-path modules 1	1 × 1 × 96 Conv (32 stride 1), 3 × 3 × 96 Conv (32 stride 1)	(224 × 224 × 64)
Normal dual-path modules 2	1 × 1 × 64 Conv (32 stride 1), 3 × 3 × 64 Conv (48 stride 1)	(224 × 224 × 80)
Dual-path reduction module 1	3 × 3 × 80 Conv (80 stride 2), 3 × 3 Max pooling (1 stride 2)	(112 × 112 × 160)
Normal dual-path modules 3	1 × 1 × 160 Conv (112 stride 1), 3 × 3 × 160 Conv (48 stride 1)	(112 × 112 × 160)
Normal dual-path modules 4	1 × 1 × 160 Conv (96 stride 1), 3 × 3 × 160 Conv (64 stride 1)	(112 × 112 × 160)
Normal dual-path modules 5	1 × 1 × 160 Conv (80 stride 1), 3 × 3 × 160 Conv (80 stride 1)	(112 × 112 × 160)
Normal dual-path modules 6	1 × 1 × 160 Conv (48 stride 1), 3 × 3 × 160 Conv (96 stride 1)	(112 × 112 × 144)
Dual-path reduction module 2	3 × 3 × 144 Conv (96 stride 2), 3 × 3 Max pooling (1 stride 2)	(56 × 56 × 240)
Normal dual-path modules 7	1 × 1 × 240 Conv (176 stride 1), 3 × 3 × 240 Conv (160 stride 1)	(56 × 56 × 336)
Normal dual-path modules 8	1 × 1 × 336 Conv (176 stride 1), 3 × 3 × 336 Conv (160 stride 1)	(56 × 56 × 336)
Dual-path reduction module 3	3 × 3 × 336 Conv (96 stride 2), Max pooling 3 × 3 (1 stride 2)	(28 × 28 × 432)
Normal dual-path modules 9	1 × 1 × 432 Conv (176 stride 1), 3 × 3 × 432 Conv (160 stride 1)	(28 × 28 × 336)
Normal dual-path modules 10	1 × 1 × 336 Conv (176 stride 1), 3 × 3 × 336 Conv (160 stride 1)	(28 × 28 × 336)
Dual-path reduction module 4	3 × 3 × 336 Conv (112 stride 2), 3 × 3 Max pooling (1 stride 2)	(14 × 14 × 448)
Normal dual-path modules 11	1 × 1 × 448 Conv (224 stride 1), 3 × 3 × 448 Conv (192 stride 1)	(14 × 14 × 416)
Normal dual-path modules 12	1 × 1 × 416 Conv (224 stride 1), 3 × 3 × 416 Conv (192 stride 1)	(14 × 14 × 416)
Dual-path reduction module 5	3 × 3 × 416 Conv (112 stride 2), 3 × 3 Max pooling (1 stride 2)	(7 × 7 × 528)
Normal dual-path modules 13	1 × 1 × 528 Conv (224 stride 1); 3 × 3 × 528 Conv (192 stride 1)	(7 × 7 × 4160
Normal dual-path modules 14	1 × 1 × 416 Conv (224 stride 1); 3 × 3 × 416 Conv (192 stride 1)	(7 × 7 × 416)
Pooling layer	7 × 7 Average pooling (1 stride 1)	(1 × 1 × 416)
Flatten	N/A	(416 × 1)
Fully connected layer	Hidden nodes (5)	(5 × 1)
Softmax layer	N/A	(5 × 1)

**Table 5:** New version of six inserted dual modules following the last normal dual module of the original CNN architecture.

Module Type	Kernel Size (Width × Height × Channel), Number and Stride	Output Size
Reduction Dual-path Module	3 × 3 × 336 Conv (96 stride 2), 3 × 3 Max pooling (1 stride 2)	(14 × 14 × 432)
Normal Dual-path Module	1 × 1 × 432 Conv (176 stride 1), 3 × 3 × 432 Conv (160 stride 1)	(14 × 14 × 336)
Normal Dual-path Module	1 × 1 × 336 Conv (176 stride 1), 3 × 3 × 336 Conv (160 stride 1)	(14 × 14 × 336)
Reduction Dual-path Module	3 × 3 × 336 Conv (96 stride 2), 3 × 3 Max pooling (1 stride 2)	(7 × 7 × 432)
Normal Dual-path Module	1 × 1 × 432 Conv (176 stride 1, 3 × 3 × 432 Conv (160 stride 1)	(7 × 7 × 336)
Normal Dual-path Module	1 × 1 × 336 Conv (176 stride 1), 3 × 3 × 336 Conv (160 stride 1)	(7 × 7 × 336)
Global Pooling Layer	7 × 7 Average pooling	(1 × 1 × 336)

**Table 6:** Comparison of highest validation accuracy of different CNNs.

CNN Architecture	Validation Accuracy (%)	Appeared Epoch	Training Time (hours/100 h)
Original Architecture	76.3068	95	N/A
Reduction Architecture	80.7823	100	6
Deepened Architecture	81.3068	93	23
Revised Deepened Architecture	81.9294	100	15
Inception Resnet v2	78.708	17	42
